# Multicenter Evaluation of QIAstat-Dx Respiratory Panel V2 for Detection of Viral and Bacterial Respiratory Pathogens

**DOI:** 10.1128/JCM.01793-19

**Published:** 2020-05-26

**Authors:** Stefan A. Boers, Willem J. G. Melchers, Cas J. A. Peters, Marga Toonen, Martin P. McHugh, Kate E. Templeton, Eric C. J. Claas

**Affiliations:** a Department of Medical Microbiology, Leiden University Medical Center, Leiden, The Netherlands; b Department of Medical Microbiology, Radboud University Medical Center, Nijmegen, The Netherlands; c Department of Medical Microbiology, Royal Infirmary of Edinburgh, Edinburgh, United Kingdom; Johns Hopkins University School of Medicine

**Keywords:** QIAstat-Dx, ePlex, molecular diagnostics, respiratory pathogen panel, respiratory tract infections, syndromic testing

## Abstract

QIAstat-Dx Respiratory Panel V2 (RP) is a novel molecular-method-based syndromic test for the simultaneous and rapid (∼70-min) detection of 18 viral and 3 bacterial pathogens causing respiratory infections. This report describes the first multicenter retrospective comparison of the performance of the QIAstat-Dx RP assay to the established ePlex Respiratory Pathogen Panel (RPP) assay, for which we used 287 respiratory samples from patients suspected with respiratory infections. The QIAstat-Dx RP assay detected 312 (92%) of the 338 respiratory targets that were detected by the ePlex RPP assay. Most of the discrepant results have been observed in the low-pathogen-load samples. In addition, the QIAstat-Dx RP assay detected 19 additional targets in 19 respiratory samples that were not detected by the ePlex RPP assay. Nine of these discordant targets were considered to represent true positives after discrepancy testing by a third method. The main advantage of the QIAstat-Dx system compared to other syndromic testing systems, including the ePlex RPP assay, is the ability to generate cycle threshold (*C_T_*) values, which could help with the interpretation of results. Taking the data together, this study showed good performance of the QIAstat-Dx RP assay in comparison to the ePlex RPP assay for the detection of respiratory pathogens. The QIAstat-Dx RP assay offers a new, rapid, and accurate sample-to-answer multiplex panel for the detection of the most common viral and bacterial respiratory pathogens and therefore has the potential to direct appropriate therapy and infection control precautions.

## Abstract (precis)

QIAstat-Dx Respiratory Panel V2 (RP) is a novel molecular-method-based syndromic test for the simultaneous and rapid (∼70-min) detection of 18 viral and 3 bacterial pathogens causing respiratory infections. This report describes the first multicenter retrospective comparison of the performance of the QIAstat-Dx RP assay to the established ePlex Respiratory Pathogen Panel (RPP) assay, for which we used 287 respiratory samples from patients suspected with respiratory infections. The QIAstat-Dx RP assay detected 312 (92%) of the 338 respiratory targets that were detected by the ePlex RPP assay.

## INTRODUCTION

Upper and lower respiratory tract infections are a major cause of hospitalization, and their morbidity and mortality result in a significant economic burden (1). A variety of respiratory pathogens, including viruses, bacteria, and fungi, can cause respiratory tract infections. As infections with these pathogens result in very similar clinical symptoms, empirical antimicrobial treatment is often initiated based on the clinical severity and epidemiological season before microbiological confirmation (2). In addition, hospital outbreaks of infections by (viral) respiratory pathogens are common and are consistently associated with significant morbidity, mortality, high pressure on isolation facilities, and increased health care costs (3, 4). Thus, the ability to rapidly and accurately diagnose respiratory tract infections is critical to ensure appropriate antimicrobial therapy and for the effective implementation of infection control measures.

Over the past 2 decades, advances in routine molecular diagnostics have revolutionized the diagnosis of respiratory tract infections. Implementation of multiplex real-time PCR (RT-PCR) assays has allowed detection of multiple respiratory pathogens in a single test with high sensitivity and specificity (5–7). One limitation is the number of fluorophores that can be differentiated by real-time PCR instruments; therefore, a maximum of five respiratory pathogens can generally be detected simultaneously in a single reaction. This means that multiple RT-PCR assays must be performed for each sample to cover the wide variety of pathogens that can cause respiratory tract infections. Recently, however, several molecular-method-based syndromic testing systems have become available that overcome this limitation. These systems can detect up to 22 respiratory pathogens in a single test with a very easy sample-to-answer workflow and a turnaround time of less than 2 h, making them suitable for decentralized or even point-of-care (POC) testing (8, 9).

Assays employing syndromic testing panels such as, for example, the BioFire FilmArray respiratory panel (RP) (bioMérieux) or ePlex respiratory pathogen panel (RPP) (GenMark) are based on endpoint detection of PCR products and therefore do not provide a quantitative indication (e.g., a cycle threshold [*C_T_*] value) corresponding to the detected respiratory pathogens. Recently, Qiagen released a novel respiratory panel (RP) for the QIAstat-Dx RT-PCR-based syndromic testing system, which does provide *C_T_* values. The QIAstat-Dx RP assay enables simultaneous testing for 21 viral and bacterial respiratory pathogens, including human adenovirus (hAdV), human bocavirus (hBoV), human coronavirus 229E (hCoV-229E), human coronavirus HKU1 (hCoV-HKU1), human coronavirus NL63 (hCoV-NL63), human coronavirus OC43 (hCoV-OC43), human metapneumovirus (hMPV), human rhinovirus/enterovirus (hRV/EV), influenza A virus, influenza A H1N1/2009 virus, influenza A H1 virus, influenza A H3 virus, influenza B virus, parainfluenza virus 1 (PIV-1), parainfluenza virus 2 (PIV-2), parainfluenza virus 3 (PIV-3), parainfluenza virus 4 (PIV-4), respiratory syncytial virus A/B (RSV-A/B), Bordetella pertussis, Legionella pneumophila, and Mycoplasma pneumoniae. All the sample preparation and analysis steps are performed automatically within disposable plastic cartridges and can be completed in 70 min.

In this study, data are presented from a retrospective multicenter evaluation of the performance of the QIAstat-Dx RP assay in clinical samples that had been submitted for the diagnosis of respiratory infections. Performance is compared to that of the ePlex RPP assay that is used as a routine diagnostic tool in the University Medical Center of Leiden (LUMC) and the University Medical Center of Nijmegen (RUMC) in the Netherlands and the Royal Infirmary of Edinburgh (RIE) in the United Kingdom.

## MATERIALS AND METHODS

### Clinical samples.

This study was performed retrospectively using 287 respiratory samples obtained from 281 patients. All 287 samples were previously submitted and prospectively tested with the ePlex RPP assay for the diagnosis of respiratory tract infections at the LUMC, the RIE, or the RUMC. Aliquots of these samples had been stored at −80°C and were available for use in a retrospective reanalysis of these samples using the QIAstat-Dx RP assay. The results obtained were compared to the initial outcome represented by the prospective ePlex RPP assay results. For this, the RUMC and RIE included selections of 98 and 101 samples, respectively, which comprised most of the targets of the QIAstat-Dx RP assay that were collected between August 2016 and November 2018. In addition, the LUMC tested a consecutive series of 88 samples from 84 patients that were submitted to this lab from January 2018 to September 2018. The 287 respiratory samples included consisted of 124 nasopharyngeal swabs (NPS) collected with an ESwab (Copan) containing 1 ml of liquid Amies media, 102 nasopharyngeal aspirates, 43 throat swabs collected with an ESwab (Copan) containing 2 ml of liquid Amies media, 14 sputum samples, and four bronchoalveolar lavage fluid samples. All sputum samples and bronchoalveolar lavage fluid samples were diluted 1:5 in phosphate-buffered saline (PBS) and homogenized by bead-beating prior to performance of the assay(s) using the ePlex RPP assay, the QIAstat-Dx RP assay, and/or laboratory-developed (multiplex) RT-PCR assays (LDTs) because of their viscosity and mucopurulent nature. No pretreatment was performed for the other types of samples. All sample processing steps took place in a biosafety cabinet. As anonymized remnant respiratory samples had been used that cannot be traced to individual patients, there was no need for approval by an ethical committee.

### ePlex RPP assay.

The ePlex RPP European CE Marking for *In Vitro* Diagnostics (CE-IVD)-cleared assay as used in this study was able to detect hAdV, hBoV, hCoV-229E, hCoV-HKU1, hCoV-NL63, hCoV-OC43, influenza A virus, influenza A H1 virus, influenza A H1N1/2009 virus, influenza A H3 virus, influenza B virus, hMPV, Middle East respiratory syndrome coronavirus (MERS-CoV), PIV-1, PIV-2, PIV-3, PIV-4, RSV-A, RSV-B, hRV/EV, B. pertussis, C. pneumoniae, L. pneumophila, and M. pneumoniae. In contrast to the FDA-cleared version of the ePlex RPP assay, the CE-IVD-cleared version of the ePlex RPP assay allows the additional detection of hBoV, MERS-CoV, B. pertussis, and L. pneumophila in respiratory samples. According to the manufacturer’s instructions, 200 μl of the respiratory sample was pipetted in a buffer tube (supplied by the manufacturer) and, after vortex mixing, transferred into the ePlex RPP test cartridge. The barcode of the test cartridge and the barcode of the corresponding sample were scanned, after which the test cartridge was inserted into an available bay of the ePlex system. The test then started automatically, and after approximately 90 min, the results of analysis of the 20 viral targets and four bacterial targets were reported as positive or not detected. If the test reported an invalid result (if, e.g., detection of the internal control [IC] failed), the samples were retested using a new test cartridge and the results of the second test were used in the data analysis.

### QIAstat-Dx RP assay.

The QIAstat-Dx RP assay was performed as described in the manufacturer’s instructions. In short, 300 μl of the respiratory sample was transferred into a QIAstat-Dx RP test cartridge. The barcode of the test cartridge and the barcode of the corresponding sample were scanned by the QIAstat-Dx operational module followed by loading of the test cartridge into the QIAstat-Dx analyzer module and starting the run. After approximately 70 min, the results from the 18 viral and 3 bacterial targets were reported as positive (with corresponding *C_T_* values) or not detected. As with ePlex RPP testing, if an invalid result was reported, the samples were retested using a new test cartridge and the results of the second test were used in the data analysis. Of note, a positive signal for the IC—regardless of the *C_T_* value—produces a valid QIAstat-Dx RP assay result. If the IC is not detected, positive results for detected and identified targets are reported to the user, but all negative results become invalid.

### Discrepant analysis.

In case of discrepant results, the discordant sample was initially retested with a new QIAstat-Dx RP and/or ePlex RPP test cartridge, thereby eliminating potential effects of prolonged sample storage. Each discordant sample that had sufficient volume available and that was not resolved by the first round of discrepant analysis was retested using the LDTs of the LUMC (for discordant samples initially tested by the LUMC and the RUMC) or RIE (for discordant samples tested by the RIE). The LDT protocols are historically based on the same assay (5, 6) but may have been updated differently, leading to minor differences (10). In short, at the LUMC, nucleic acids were extracted from 200 μl of respiratory samples and eluted in a 100-μl volume using a MagNa Pure 96 instrument (Roche). Ten microliters of the nucleic acid extracts was then tested using real-time PCR assays that were performed on a Bio-Rad CFX96 real-time PCR instrument (Bio-Rad). At the RIE, the LDT methods consisted of the extraction of nucleic acids from 200 μl of respiratory samples that were subsequently eluted in 100 μl using a NucliSENS easyMAG system (bioMérieux). Real-time PCR amplification and detection were then performed using 3.5 to 4.5 μl of the nucleic acid extracts and an ABI 7500 fast thermocycler (Applied Biosystems). The LDTs performed at both laboratories were designed to detect a variety of viral and bacterial respiratory pathogens with updated versions (if necessary) of primers and probes as previously described (5–7, 11–14). All LDTs have been implemented for routine diagnostic use after validation performed according to the ISO 15189:2012 guideline for clinical laboratories.

## RESULTS

A total of 287 respiratory samples from 281 patients were included in this study. These samples included 124 NPS samples, which is the intended-use sample number specified for both the QIAstat-Dx RP assay and the ePlex RPP assay (in the FDA-cleared version and the CE-IVD-marked version of both products). Additionally, 163 non-NPS samples were used (Table 1). A total of 222 of these 287 samples tested positive for at least one respiratory pathogen using the ePlex RPP assay, and no respiratory pathogen was detected in the remaining 65 samples. Of the 287 samples tested, 13 (5%) and 14 (5%) samples had invalid ePlex RPP and QIAstat-Dx RP results, respectively (Table 1). All samples with an initial invalid result generated a valid result upon retesting using the corresponding method.

**TABLE 1:** Comparison of results of respiratory pathogen detection by the ePlex RPP assay and the QIAstat-Dx RP assay by sample type^*a*^

Sample type	Laboratory	No. of samples	No. of results				No. of initial assay failures	
			ePlex^+^/QIAstat-Dx^+^	ePlex^+^/QIAstat-Dx^−^	ePlex^−^/QIAstat-Dx^+^	ePlex^−^/QIAstat-Dx^−^	ePlex RPP	QIAstat-Dx RP
NPS	LUMC	26	14			532	1	2
	RUMC	98	115	4	5	1,934		2

NA	LUMC	9	8	2		179	1	
	RIE	93	137	16	11	1,789	8	6

TS	LUMC	35	22			713	1	3
	RIE	8	7	2	1	158		

Sputum	LUMC	14	9	2	2	281	2	1

BAL	LUMC	4				84		

a BAL, bronchoalveolar lavage fluid; LUMC, Leiden University Medical Center; NA, nasopharyngeal aspirate; NPS, nasopharyngeal swab; RIE, Royal Infirmary of Edinburgh; RP, respiratory panel; RPP, respiratory pathogen panel; RUMC, Radboud University Medical Center; TS, throat swab.

The performance characteristics for individual QIAstat-Dx RP targets are presented in Table 2 (see also Tables S1 to S3 in the supplemental material). The overall level of agreement for the QIAstat-Dx RP assay with targets detected by the ePlex RPP assay was shown to be 312 of the 338 targets (92%). For individual targets, the concordance with the ePlex RPP assay was 100% for hCoV-HKU1 (7/7), hCoV-NL63 (9/9), influenza A-H3 virus (20/20), PIV-1 (3/3), PIV-2 (3/3), PIV-3 (4/4), B. pertussis (2/2), and M. pneumoniae (5/5) and ≥95% for hMPV (26/27), influenza A virus (36/38), and RSV-A/B (41/43). No complete concordance was observed for hAdV, for which 17 of 19 positives (17/19) were detected by the QIAstat-Dx RP assay in comparison to the ePlex RPP assay, or for hBoV (17/20), hCoV-OC43 (10/11), hRV/EV (56/64), influenza A-H1/2009 virus (12/14), influenza B virus (40/44), or PIV-4 (4/5). The results determined with respect to detection of respiratory pathogens in samples containing a single respiratory pathogen were concordant in 153/160 (96%) samples. For samples containing multiple respiratory pathogens, the same respiratory pathogens that were detected by the ePlex RPP assay were also identified by the QIAstat-Dx RP assay in 36/46, 7/12, 2/3, and 1/1 in cases of two, three, four, and five respiratory pathogens present, respectively.

**TABLE 2:** Comparison of results of respiratory pathogen detection by the ePlex RPP assay and the QIAstat-Dx RP assay

QIAstat-Dx RP target	No. of results			
	ePlex^+^/QIAstat-Dx^+^	ePlex^+^/QIAstat-Dx^−^	ePlex^−^/QIAstat-Dx^+^	ePlex^−^/QIAstat-Dx^−^
Viruses				
Human adenovirus	17	2	2	266
Human bocavirus	17	3	1	266
Human coronavirus 229E			1	286
Human coronavirus HKU1	7		3	277
Human coronavirus NL63	9		1	277
Human coronavirus OC43	10	1	1	275
Human metapneumovirus A/B	26	1		260
Human rhinovirus/enterovirus	56	8	3	220
Influenza A virus	36	2	1	248
Influenza A H1N1/2009 virus	12	2		273
Influenza A H1 virus				287
Influenza A H3 virus	20		1	266
Influenza B virus	40	4		243
Parainfluenza virus 1	3		2	282
Parainfluenza virus 2	3			284
Parainfluenza virus 3	4		2	281
Parainfluenza virus 4	4	1	1	281
Respiratory syncytial virus A/B^*a*^	41	2		244

Bacteria				
Bordetella pertussis	2			285
Legionella pneumophila				287
Mycoplasma pneumoniae	5			282

Total	312	26	19	5,670

a The QIAstat-Dx Respiratory Panel (RP) assay does not differentiate between respiratory syncytial virus (RSV) A and RSV-B. In total, 13 RSV-A and 30 RSV-B were detected using the ePlex Respiratory Pathogen Panel (RPP) assay.

**FIG 1:**
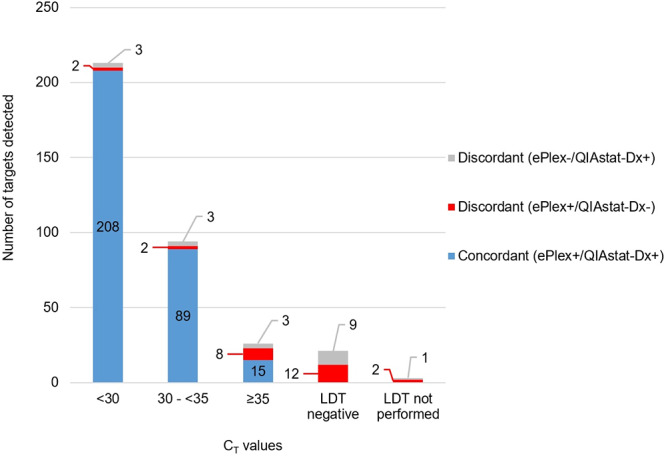
Comparison of results of respiratory pathogen detection by the ePlex RPP assay and the QIAstat-Dx RP assay determined by *C_T_* value. Concordant results are grouped based on the *C_T_* values obtained with the QIAstat-Dx RP assay, while discordant results are grouped based on the *C_T_* values obtained with LDTs as part of the discrepant analysis. No discrepant testing was performed for three discordant target results because there was not enough sample volume available for additional tests (*n *= 1) or because no LDT was available to detect the discordant target at the specific laboratory (*n *= 2).

As shown in Fig. 1 and Fig. S1 to S3 in the supplemental material, a total of 26 discordant results (ePlex positive/QIAstat-Dx negative [ePlex^+^/QIAstat-Dx^−^]) were obtained from 26 respiratory samples. Twelve of these 26 discordant targets were confirmed by discrepant testing using laboratory-developed (multiplex) RT-PCR assays (LDTs), indicating that these discrepant target results represent true positives (see Table S4 in the supplemental material). These included two targets with a *C_T_* value of less than 30 (hMPV and influenza B virus), two targets with *C_T_* values between 30 and 35 (hBoV and influenza B virus), and eight targets with a *C_T_* value of 35 or higher (hAdV, hBoV [2×], hCoV-OC43, hRV/EV [2×], influenza A virus, and influenza B virus). The other 14 discordant targets either tested negative by discrepant testing (*n *= 12) or were not included for discrepant testing as no subtyping of influenza virus was performed at the specific laboratory (*n *= 2). Those two influenza A H1N1/2009-positive samples were both detected as influenza A virus by the QIAstat-Dx RP assay.

In comparison with the QIAstat-Dx RP assay, the ePlex RPP assay contains two additional respiratory targets (i.e., Middle East respiratory syndrome coronavirus [MERS-CoV] and Chlamydophila pneumoniae) and includes differentiation of RSV subtypes. This resulted in the additional detection of C. pneumoniae in one respiratory sample—which also tested positive for hRV/EV and RSV(-B) by both methods—using the ePlex RPP assay. This result was confirmed by an additional LDT performed with a *C_T_* value of 32.6 (Table S4). Furthermore, a total of 13 RSV-A and 30 RSV-B targets were detected using the ePlex RPP assay, while 41 RSV-A/B targets were detected by the QIAstat-Dx RP assay. The two discrepant RSV targets consisted of one RSV-A target and one RSV-B target, both of which tested negative by discrepancy testing using the LDT.

The QIAstat-Dx RP assay detected 19 additional targets in 19 samples that were not detected by the ePlex RPP assay (ePlex^−^/QIAstat-Dx^+^). After resolution by discrepancy testing using LDT, 9 of these 19 discordant targets were confirmed by LDT and considered to be true positives (Fig. 1; see also Table S4). These included three targets with LDT-based *C_T_* values lower than 30 (i.e., hCoV-HKU1, hCoV-229E, and hBoV), three targets with *C_T_* values between 30 and 35 (i.e., hRV/EV, influenza A H3 virus [detected as influenza A virus by the ePlex RPP assay], and PIV-1), and three targets with *C_T_* values higher than 35 (i.e., hCoV-HKU1, hCoV-OC43, and hAdV). The remaining 10 discordant targets either tested negative by LDT (*n *= 9) or could not be resolved as there was not enough sample volume available for additional tests (*n *= 1).

Sixty-four of the 65 samples with a negative ePlex RPP assay result tested negative with the QIAstat-Dx RP assay as well. The discordant sample tested positive for hCoV-HKU1 with the QIAstat-Dx RP assay, and that result was confirmed by discrepant testing with a *C_T_* value of 25.4. In contrast, 7 of the 222 samples with a positive ePlex RPP assay result tested negative with the QIAstat-Dx RP assay. These included four samples that tested positive for influenza B virus (three of four of these positives were confirmed by discrepant testing with *C_T_* values of 21.5, 34.1, and 38.4, respectively), one sample positive for hRV/EV (confirmed with a *C_T_* value of 35.4), and one sample each that tested positive for influenza A virus and RSV-B that could not be confirmed by discrepant testing. The discordant influenza B virus-positive sample with the highest viral load (i.e., with a *C_T_* value of 21.5) did test positive for influenza B virus in the QIAstat-Dx RP assay with a *C_T_* value of 29.4 after a 100-fold dilution of the sample in phosphate-buffered saline (PBS) was performed.

## DISCUSSION

In this multicenter study, the performance characteristics of the new QIAstat-Dx RP assay on the Qiagen QIAstat-Dx system (formerly STAT-Dx; DiagCORE) were examined by measuring agreement with the results of the ePlex RPP assay, both being commercially available multiplex pathogen panels for diagnosing respiratory tract infections. For this, 287 respiratory samples that were previously tested for the presence of respiratory pathogens with the ePlex RPP assay were retested in this study with the QIAstat-Dx RP assay. Although the QIAstat-Dx RP assay had been FDA and CE-IVD cleared for detection of respiratory pathogens from NPS samples only, a range of alternative sample types that are also regularly submitted for diagnosis of respiratory pathogens were included in this study.

The QIAstat-Dx RP assay detected 129 of the 133 respiratory targets (97%) and 183 of the 205 respiratory targets (89%) in NPS and non-NPS samples, respectively, that were detected by the ePlex RPP assay. Twelve of the 26 discordant targets were confirmed by LDT with generally high *C_T_* values, indicating that the discrepant results can be explained by their low viral loads. However, two discordant targets, i.e., hMPV with an LDT-based *C_T_* value of 23.6 and influenza B virus with an LDT-based *C_T_* value of 21.5, likely represented the result of partial or primer-specific PCR inhibition effects. For example, the “valid” internal control (IC) *C_T_* value of 36.1 measured in the hMPV-positive sample with the QIAstat-Dx RP assay is considerably higher than the median IC *C_T_* value of 33.3 (± 2.2) that we measured in this study. In addition, the remaining influenza B virus discrepant sample—with a valid IC *C_T_* value of 33.5—did test positive for influenza B virus after retesting of a 100-fold dilution of the corresponding sputum sample. In our opinion, the reliability of results can be increased by setting stricter inhibition criteria of, for example, three *C_T_* values above the median IC value, as this already indicates an IC reaction that is 10-fold less efficient. In 18 different samples, the QIAstat-Dx RP assay identified 18 respiratory pathogens that were not detected by the ePlex RPP assay (ePlex^−^/QIAstat-Dx^+^). In addition, one nonsubtyped influenza A virus detected by the ePlex RPP assay was subtyped as influenza A H3 virus by the QIAstat-Dx RP assay. One of the selected negative samples was shown to contain an hCoV-HKU1 strain with an LDT-based *C_T_* value of 25.4, while all other ePlex^−^/QIAstat-Dx^+^ pathogens were detected as representing coinfections corresponding to at least one (other) respiratory pathogen. Discrepant analysis revealed that 6 of the 19 additional targets were considered to be true positives, with *C_T_* values lower than 35. However, since the ePlex RPP assay does not provide *C_T_* values for the IC used and the targets that it detects, it remains unclear if these six ePlex^−^/QIAstat-Dx^+^ results were the result of PCR inhibition within the ePlex RPP test cartridge or the result of a more efficient detection of polymicrobial respiratory infections by the QIAstat-Dx RP assay.

The QIAstat-Dx RP assay (with a 95% success rate on the initial test attempt) is as reliable as the ePlex RPP assay and offers a similar sample-to-answer workflow with minimal hands-on time and a rapid turnaround time to results. The main advantage of the QIAstat-Dx RP assay over the ePlex RPP assay (and other syndromic testing systems), however, is the ability to generate *C_T_* values that can help with the interpretation of results. In addition, the QIAstat-Dx RP test cartridge has two sample ports and can accommodate both liquid transfer and direct swab processing. The direct processing of swabs using the QIAstat-Dx RP assay is faster and easier than processing of liquid transfers. This was not tested in the current study as this option is not present in the ePlex cartridge. The ePlex RPP assay, on the other hand, contains more respiratory targets that enable the additional detection of C. pneumoniae and MERS-CoV and discrimination between RSV-A and RSV-B subtypes. Furthermore, previous studies have demonstrated that rapid diagnosis of respiratory infections by implementation of syndromic testing panels, such as the QIAstat-Dx RP and ePlex RPP assays, can lead to decreased length of stay, improved antimicrobial stewardship, and a reduction in the number of days that patients were kept in isolation (2, 15). Several other sample-to-answer systems are now commercially available, such as the ID NOW (formerly Alere I, now Abbott), cobas Liat (Roche), and Xpert Xpress (Cepheid) systems, which can even deliver test results within 15 to 30 min. However, these ultrafast assays are limited to detection of a maximum of three respiratory pathogens, including influenza A and B viruses and RSV (16–19).

This study had its limitations. First, in cases of discrepant results, discordant samples were retested using slightly different LDTs at the LUMC or the RIE. Although the performances of these assays are regularly evaluated according to the ISO 15189:2012 guideline for clinical laboratories, variations in sensitivity and specificity may have affected the accuracy of the data determined with the resolved discordant samples (i.e., determination of false-positive and false-negative results). Second, the total number of respiratory samples positive for hCoV-229 (*n *= 0), influenza A H1 virus (*n *= 0), PIV-1 (*n *= 3), PIV-2 (*n *= 3), PIV-3 (*n *= 4), PIV-4 (*n *= 4), B. pertussis (*n *= 2), L. pneumophila (*n *= 0), and M. pneumoniae (*n *= 5) was too low for a proper assessment of the QIAstat-Dx RP assay.

In conclusion, the QIAstat-Dx RP assay offers a large panel of both viral and bacterial respiratory pathogens in a simple sample-to-answer format with minimal hands-on time and a rapid turnaround time to results. The assay performance was equivalent to that of the ePlex RPP assay, which previously showed excellent performance compared to LDTs for diagnosing respiratory tract infections (9). Therefore, the QIAstat-Dx RP assay represents a new and good candidate for the detection of respiratory pathogens in either a laboratory or a decentralized setting using a syndromic approach.

## Supplementary Material

Supplemental file 1
