# Supplementary material for: Multicenter Evaluation of QIAstat-Dx Respiratory Panel V2 for Detection of Viral and Bacterial Respiratory Pathogens
Source: J Clin Microbiol. 2020 May 26;58(6):e01793-19. doi: 10.1128/JCM.01793-19 (PMC7269373; doi:10.1128/JCM.01793-19)
Supplement: Supplemental file 1 [file JCM.01793-19-s0001.pdf]

TABLE S1 Comparison of respiratory pathogen detection by the ePlex® RPP assay and the QIAstat-Dx® RP assay at the Leiden University Medical Center (LUMC).

	No. of results			
QIAstat-Dx® RP targets	ePlex®+/QIAstat-Dx®+	ePlex®+/QIAstat-Dx®−	ePlex®−/QIAstat-Dx®+	ePlex®−/QIAstat-Dx®−
Viruses				
Human adenovirus				88
Human bocavirus				88
Human coronavirus 229E			1	87
Human coronavirus HKU1	2		1	85
Human coronavirus NL63	1			87
Human coronavirus OC43	1			87
Human metapneumovirus A/B	6			82
Human rhinovirus/enterovirus	10	2		76
Influenza A virus	9			79
Influenza A H1N1/2009 virus	5	1		82
Influenza A H1 virus				88
Influenza A H3 virus	2			86
Influenza B virus	13	1		74
Parainfluenza virus 1				88
Parainfluenza virus 2				88
Parainfluenza virus 3	1			87
Parainfluenza virus 4				88
Respiratory syncytial virus A/B[A]	3			85
Bacteria				
Bordetella pertussis				88
Legionella pneumophila				88
Mycoplasma pneumoniae				88
Total	53	4	2	1,789

[A] The QIAstat-Dx® Respiratory Panel (RP) assay does not differentiate between respiratory syncytial virus (RSV) A and RSV-B. In total, 3 RSV-B were detected using the ePlex® Respiratory Pathogen Panel (RPP) assay.

TABLE S2 Comparison of respiratory pathogen detection by the ePlex® RPP assay and the QIAstat-Dx® RP assay at the Radboud University Medical Center (RUMC).

	No. of results			
QIAstat-Dx® RP targets	ePlex®+/QIAstat-Dx®+	ePlex®+/QIAstat-Dx®−	ePlex®−/QIAstat-Dx®+	ePlex®−/QIAstat-Dx®−
Viruses				
Human adenovirus	3			95
Human bocavirus	7		1	90
Human coronavirus 229E				98
Human coronavirus HKU1	3		2	93
Human coronavirus NL63	2			96
Human coronavirus OC43	1			97
Human metapneumovirus A/B	7			91
Human rhinovirus/enterovirus	23	2	1	72
Influenza A virus	12		1	85
Influenza A H1N1/2009 virus	5	1		92
Influenza A H1 virus				98
Influenza A H3 virus	5			93
Influenza B virus	24	1		73
Parainfluenza virus 1	1			97
Parainfluenza virus 2				98
Parainfluenza virus 3	2			96
Parainfluenza virus 4	1			97
Respiratory syncytial virus A/B[A]	16			82
Bacteria				
Bordetella pertussis	2			96
Legionella pneumophila				98
Mycoplasma pneumoniae	1			97
Total	115	4	5	1,934

[A] The QIAstat-Dx® Respiratory Panel (RP) assay does not differentiate between respiratory syncytial virus (RSV) A and RSV-B. In total, 1 RSV-A and 15 RSV-B were detected using the ePlex® Respiratory Pathogen Panel (RPP) assay.

TABLE S3 Comparison of respiratory pathogen detection by the ePlex® RPP assay and the QIAstat-Dx® RP assay at the Royal Infirmary of Edinburgh (RIE).

	No. of results			
QIAstat-Dx® RP targets	ePlex®+/QIAstat-Dx®+	ePlex®+/QIAstat-Dx®−	ePlex®−/QIAstat-Dx®+	ePlex®−/QIAstat-Dx®−
Viruses				
Human adenovirus	14	2	2	83
Human bocavirus	10	3		88
Human coronavirus 229E				101
Human coronavirus HKU1	2			99
Human coronavirus NL63	6		1	94
Human coronavirus OC43	8	1	1	91
Human metapneumovirus A/B	13	1		87
Human rhinovirus/enterovirus	23	4	2	72
Influenza A virus	15	2		84
Influenza A H1N1/2009 virus	2			99
Influenza A H1 virus				101
Influenza A H3 virus	13		1	87
Influenza B virus	3	2		96
Parainfluenza virus 1	2		2	97
Parainfluenza virus 2	3			98
Parainfluenza virus 3	1		2	98
Parainfluenza virus 4	3	1	1	96
Respiratory syncytial virus A/B[A]	22	2		77
Bacteria				
Bordetella pertussis				101
Legionella pneumophila				101
Mycoplasma pneumoniae	4			97
Total	144	18	12	1,947

[A] The QIAstat-Dx® Respiratory Panel (RP) assay does not differentiate between respiratory syncytial virus (RSV) A and RSV-B. In total, 12 RSV-A and 12 RSV-B were detected using the ePlex® Respiratory Pathogen Panel (RPP) assay.

TABLE S4 Discrepant analysis results.

Sample	ePlex® RPP assay result	QIAstat-Dx® RP assay result (C_T value)	LDT assay result (C_T value)
Discordant results obtained from respiratory samples tested at LUMC			
1_NA	hCoV-NL63; **hRV/EV**	hCoV-NL63 (19.1)	hRV/EV not detected
2_NA	hCoV-HKU1; **hRV/EV**	hCoV-HKU1 (34.6)	hRV/EV not detected
3_S	Flu-A; **Flu-A-H1/2009**	Flu-A	Not performed[A]
4_S	RSV-B	RSV-A/B (14.8); **hCoV-HKU1 (32.2)**	hCoV-HKU1 (27.4)
5_S	**Flu-B**	No pathogens detected[C]	Flu-B (21.5)
6_S	No pathogens detected	**hCoV-229E (31.4)**	hCoV-229E (25.4)
Discordant results obtained from respiratory samples tested at RUMC			
7_NPS	**hRV/EV**	No pathogens detected	hRV/EV (35.4)[E]
8_NPS	Flu-A; Flu-**A-H1/2009**	Flu-A (31.7)	Not performed[A]
9_NPS	**Flu-B**	No pathogens detected	Flu-B (38.4)[E]
10_NPS	Flu-B	Flu-B (17.5); **hCoV-HKU1 (33.6)**	hCoV-HKU1 (36.0)[E]
11_NPS	Flu-B; **hRV/EV**	Flu-B (20.6)	hRV/EV not detected[E]
12_NPS	Flu-B	Flu-B (31.8); **hCoV-HKU1 (33.5)**	hCoV-HKU1 not detected[E]
13_NPS	hRV/EV	hRV/EV (27.1); **Flu-A (36.8)**	Not performed[B]
14_NPS	Flu-B	Flu-B (26.8); **hRV/EV (31.0)**	hRV/EV (32.5)[E]
15_NPS	hRV/EV; PIV-3	hRV/EV (17.8); PIV-3 (26.7); **hBoV (33.5)**	hBoV (29.9)[E]
Discordant results obtained from respiratory samples tested at RIE			
16_NA	hRV/EV; hAdV	hRV/EV (19.6); hAdV (25.5); **PIV-3 (36.0)**	PIV-3 not detected
17_NA	**hRV/EV**; *M. pneumoniae*	*M. pneumoniae* (22.1)	hRV/EV not detected
18_NA	hRV/EV	hRV/EV (22.9); **hAdV (34.1)**	hAdV not detected
19_NA	RSV-B	RSV-A/B (17.3); **hCoV-OC43 (34.6)**	hCoV-OC43 (39.1)
20_NA	Flu-A	Flu-A (35.0); **Flu-A-H3 (34.3)**	Flu-A-H3 (32.9)
21_NA	Flu-A; Flu-A-H3	Flu-A (26.0); Flu-A-H3 (26.4); **PIV-3 (33.3)**	PIV-3 not detected
22_NA	**Flu-A**	No pathogens detected	Flu-A not detected
23_NA	RSV-A	RSV-A/B (17.3); **PIV-1 (32.7)**	PIV-1 (31.4)
24_NA	**hMpV**; hBoV	hBoV (21.8)	hMpV (23.6)
25_NA	hMpV; hBoV; **hAdV**	hMpV (20.7); hBoV (25.6); **hRV/EV (32.7)**	hAdV (37.7);<br>hRV/EV not detected
26_NA	**RSV-B**	No pathogens detected	RSV-B not detected
27_NA	RSV-B; **hRV/EV**	RSV-A/B (14.1); **PIV-1 (37.3)**	hRV/EV and PIV-1 not detected
28_NA	**Flu-A**; Flu-A-H3	Flu-A-H3 (37.6)	Flu-A (36.1)
29_NA	RSV-A; **hAdV**	RSV-A/B (16.5)	hAdV not detected

30_NA	hAdV; RSV-A; hRV/EV	hAdV (31.6); RSV-A/B (18.0); hRV/EV (31.9); **PIV-4 (34.7)**	PIV-4 not detected
31_NA	hCoV-OC43	hCoV-OC43 (29.8); **hRV/EV (33.6)**	hRV/EV not detected
32_NA	hAdV; hRV/EV; **hBoV**	hAdV (16.6); hRV/EV (29.2)	hBoV (30.3)
33_NA	hAdV; hMpV; **hRV/EV**	hAdV (32.6); hMpV (31.4)	hRV/EV not detected
34_NA	**hRV/EV**; Flu-A; Flu-A-H3; hBoV	**hAdV (31.2)**; Flu-A (25.2); Flu-A-H3 (25.2); hBoV (29.7)	hRV/EV (36.9); hAdV (39.0)
35_NA	hAdV; hBoV; hRV/EV; **RSV-A**	hAdV (13.6); hBoV (33.1); hRV/EV (31.6)	RSV-A not detected
36_NA	*M. pneumoniae*; **hBoV**	*M. pneumoniae* (30.0)	hBoV (35.8)
37_NA	**PIV-4**; hRV/EV	hRV/EV (28.5)	PIV-4 not detected
38_NA	**hCoV-OC43**; RSV-B	RSV-A/B (19.3)	hCoV-OC43 (38.3)
39_NA	hCoV-OC43; hRV/EV; **hBoV**	hCoV-OC43 (25.2); hRV/EV (29.4)	hBoV (35.1)
40_NA	hRV/EV; RSV-B; ***C. pneumoniae***[D]	hRV/EV; RSV-A/B	*C. pneumoniae* (32.6)
41_TS	**Flu-B**	No pathogens detected	Flu-B not detected
42_TS	**Flu-B**	No pathogens detected	Flu-B (34.1)
43_TS	hAdV; hRV/EV	hAdV (16.3); hRV/EV (30.3); **hCoV-NL63 (36.2)**	hCoV-NL63 not detected

Samples were derived from nasopharyngeal swabs (NPS), nasopharyngeal aspirates (NA), throat swabs (TS), sputum (S), and bronchoalveolar lavage fluids (BAL). Respiratory pathogens in bold were only detected by one of the two methods used, either the ePlex® Respiratory Pathogen panel (RPP) assay or the QIAstat-Dx® Respiratory Panel (RP) assay. Discrepant testing was performed by using laboratory developed (multiplex) real-time PCR assay (LDTs) at the corresponding laboratory or elsewhere as indicated.

[A] Discrepant analysis was not performed because there was no LDT available that could detect these targets at the specific laboratory.

[B] Discrepant analysis was not performed because there was not enough sample volume available.

[C] A 100-fold dilution of the corresponding sputum sample tested positive for influenza B virus by the QIAstat-Dx® RP assay with a C_T value of 29.4.

[D] *C. pneumoniae* is not part of the QIAstat-Dx® RP assay.

[E] Discrepant testing was performed by using the LDTs of the LUMC.

Flu – influenza virus; hAdV – human adenovirus; hBoV – human bocavirus; hCoV – human coronavirus; hMPV – human metapneumovirus; hRV/EV – human rhinovirus/enterovirus; LUMC – Leiden University Medical Center; PIV – parainfluenza virus; RIE – Royal Infirmary of Edinburgh; RSV – respiratory syncytial virus; RUMC – Radboud University Medical Center.

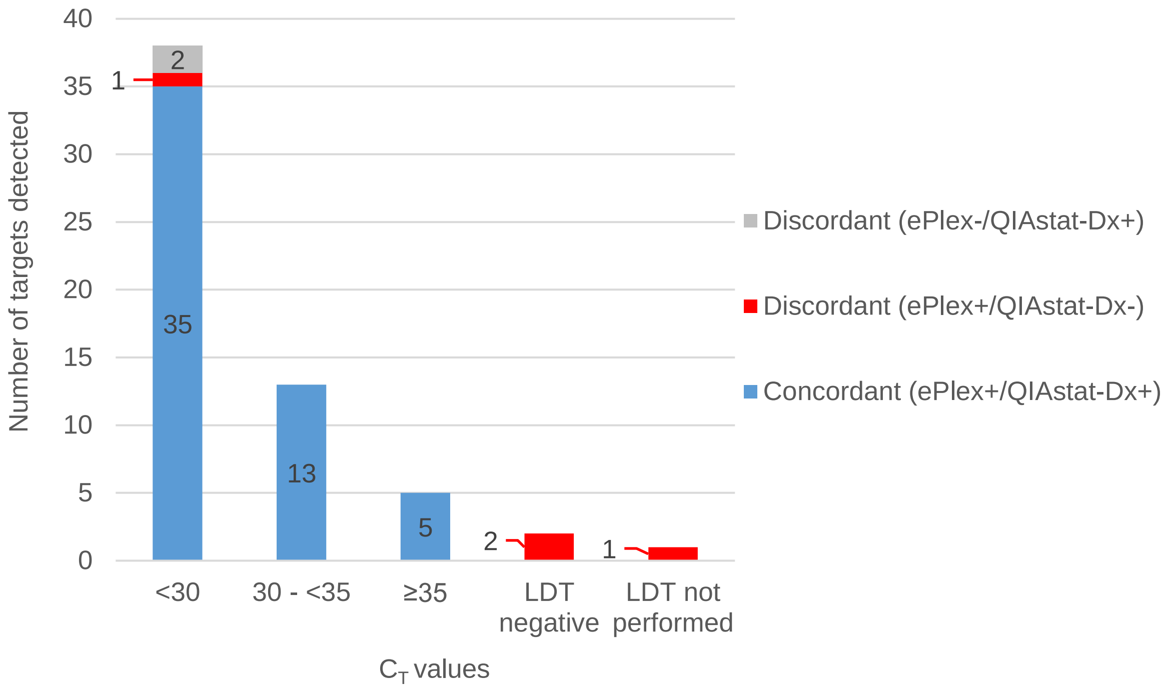

FIG S1 Comparison of respiratory pathogen detection by the ePlex® RPP assay and the QIAstat-Dx® RP assay by C_T value at the Leiden University Medical Center (LUMC). Concordant results are grouped based on the C_T values obtained with the QIAstat-Dx® RP assay, while discordant results are grouped based on the C_T values obtained with LDTs performed at the LUMC as part of the discrepant analysis.

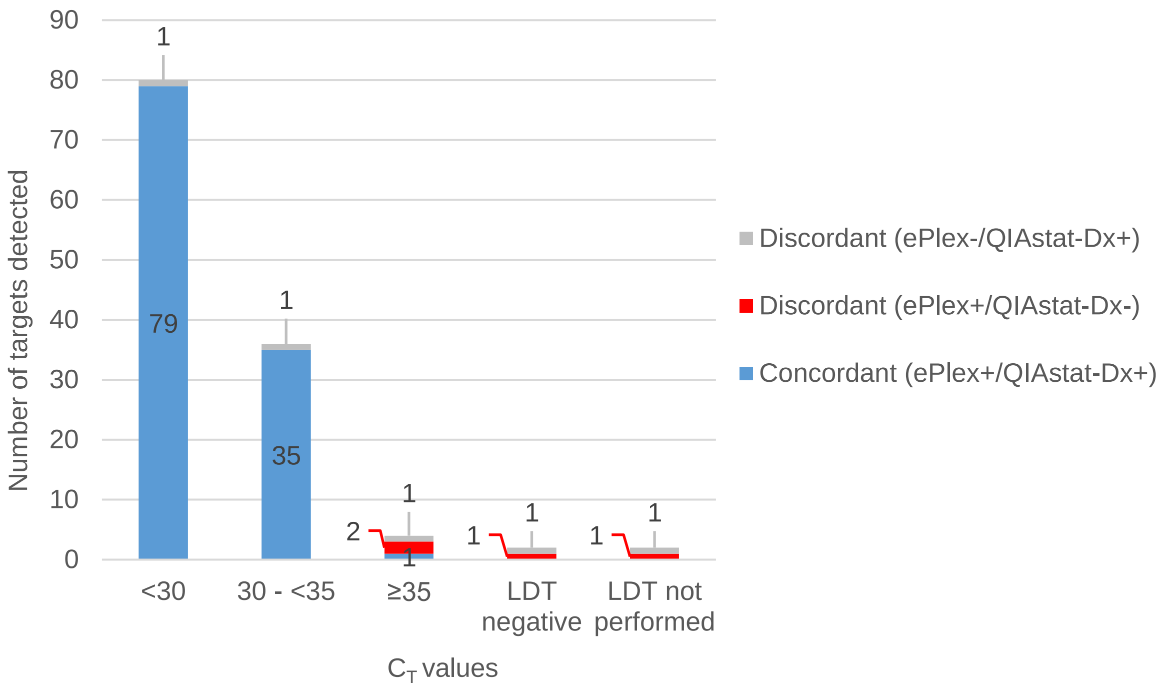

FIG S2 Comparison of respiratory pathogen detection by the ePlex® RPP assay and the QIAstat-Dx® RP assay by C_T value at the Radboud University Medical Center (RUMC). Concordant results are grouped based on the C_T values obtained with the QIAstat-Dx® RP assay, while discordant results are grouped based on the C_T values obtained with LDTs performed at the Leiden University Medical Center (LUMC) as part of the discrepant analysis.

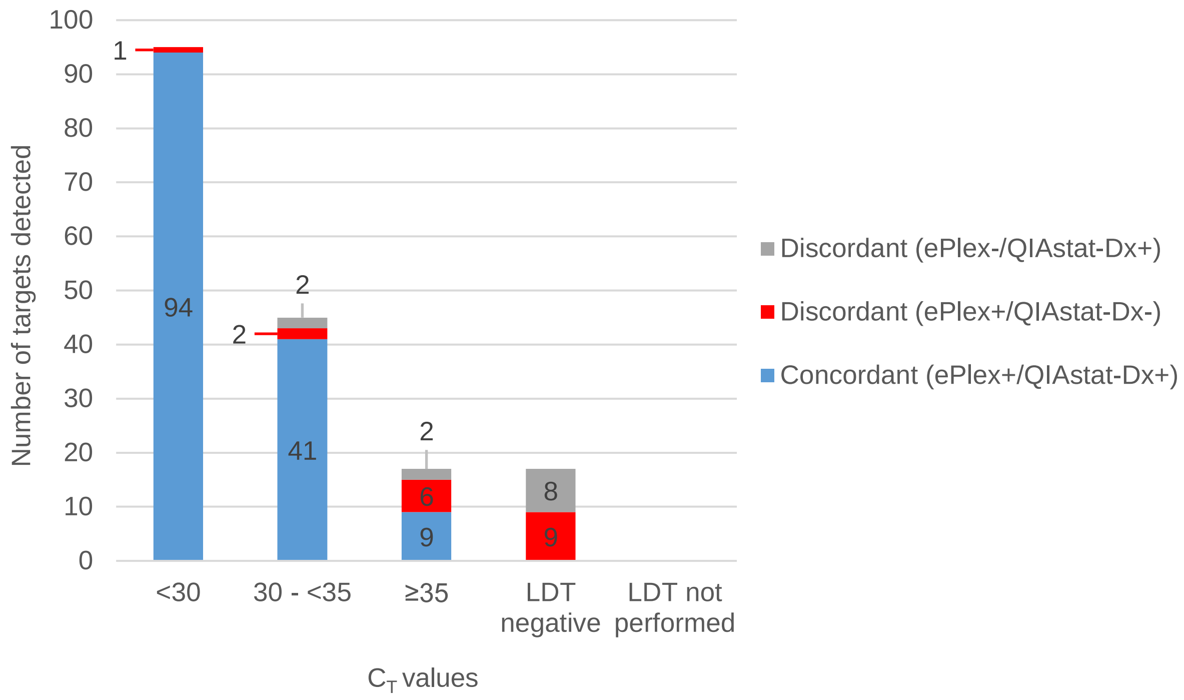

FIG S3 Comparison of respiratory pathogen detection by the ePlex® RPP assay and the QIAstat-Dx® RP assay by C_T value at the Royal Infirmary of Edinburgh (RIE). Concordant results are grouped based on the C_T values obtained with the QIAstat-Dx® RP assay, while discordant results are grouped based on the C_T values obtained with LDTs performed at the RIE as part of the discrepant analysis.
